# Outcomes of Treatment for Central Serous Chorioretinopathy With Half-Fluence, Half-Dose Photodynamic Therapy

**DOI:** 10.1177/24741264261444276

**Published:** 2026-04-27

**Authors:** Amit V. Mishra, Deven M. Deonarain, Graeme K. Loh, Matthew T.S. Tennant

**Affiliations:** 1 Dalhousie University, Department of Ophthalmology and Visual Sciences, Halifax, Canada; 2 University of Alberta, Faculty of Medicine, Edmonton, Canada; 3 Greenlane Clinical Center, Department of Ophthalmology, Auckland, New Zealand; 4 University of Alberta, Department of Ophthalmology and Visual Sciences, Edmonton, Canada; 5 Alberta Retina Consultants, Edmonton, Canada

**Keywords:** central serous chorioretinopathy, choroid, retina

## Abstract

**Purpose:** To determine outcomes in eyes with central serous chorioretinopathy (CSCR) treated with half-half photodynamic therapy (PDT). **Methods:** This retrospective cohort review included patients with CSCR treated with half-half PDT at Alberta Retina Consultants between July 2007 and July 2023. Exclusion criteria included concomitant ocular disease or history of ocular surgery, intraocular injection, or laser therapy in the treated eye. The primary outcome was posttreatment visual acuity change. Secondary outcomes included central subfield thickness (CST) and fluid resolution assessed at 3 months, in addition to fluid recurrence, need for retreatment, and reports of adverse events at any timepoint after PDT. **Results:** Three hundred and seventy-eight eyes were included, with a mean time to treatment from presentation of 4.6 months. There was a statistically significant visual gain of 4.4 Early Treatment Diabetic Retinopathy Study letters with treatment (*P* < .05). Complete resolution of fluid was seen in 56.8% of eyes 3 months’ posttreatment. Complete resolution at 6 months was seen in 64.0% of eyes. Recurrence was seen in 16.7% of eyes. There were no cases of macular atrophy secondary to PDT. Over the study period, pachychoroid neovasculopathy was identified in 3.4% of treated eyes. **Conclusions:** Half-half PDT for active CSCR yielded improvements in both best-corrected visual acuity and CST. Half-half PDT is a safe alternative treatment option that is slightly less effective than half-dose, full-fluence PDT.

## Introduction

Central serous chorioretinopathy (CSCR) is a retinal disorder characterized by macular neurosensory detachment and pigment epithelial detachment (PED) due to the accumulation of subretinal fluid (SRF).^1^ Associated changes in morphology and function of the retinal pigment epithelium (RPE) and choroidal vasculature are implicated in the development of CSCR.^2^ Patients typically present with decreased visual acuity (VA) in association with central scotoma, metamorphopsia, dyschromatopsia, micropsia, and reduced contrast sensitivity. CSCR predominantly affects men, with estimated annual incidences of 9.9 per 100 000 men and 1.7 per 100 000 women, with a peak age of onset of approximately 45 years of age.^1,3^ As CSCR commonly affects people of working age, the impacts on quality of life are unique in comparison to other retinal diseases, such as age-related macular degeneration. Risk factors for CSCR include exogenous steroid use, endogenous hypercortisolism, hypertension, psychologic stress, sleep apnea, phosphodiesterase-5 inhibitor use, and pregnancy.^4^

Although the exact etiology of CSCR has not been elucidated, suggested pathophysiologic mechanisms include hyperpermeability of choroidal vessels secondary to vascular stasis and steroidal activation along with dysfunction of RPE membrane pumps, resulting in accumulation of SRF.^1,4^ The disease can present in an acute or chronic form based on the duration of serous neurosensory detachment. Most cases of acute CSCR will resolve spontaneously, although recurrence after resolution is not uncommon. A large population-based study in the United States with 22 years of follow-up identified a 31% rate of CSCR recurrence, with a median time to recurrence of 1.3 years. Chronic CSCR tends to occur in patients over the age of 50 and is associated with poorer long-term visual outcomes and higher rates of choroidal neovascularization (NV).^5,6^

Management of CSCR includes observation or laser therapies (photodynamic therapy [PDT], laser photocoagulation, and subthreshold micropulse laser), with the use of intravitreal (IVT) agents and systemic therapies occurring less commonly.^6^ PDT has become a mainstay of CSCR treatment and involves the intravenous administration of the photosensitive agent verteporfin at a dose of 6 mg/m^2^ of body surface area followed by activation via delivery of a specific wavelength (689 nm) of nonthermal laser light at an intensity of 50 J/cm^2^.^7^ Verteporfin accumulates in the choroidal vasculature and, after photoactivation, reactive oxygen species are produced that induce cellular apoptosis and closure of choroidal vessels.^7^ Adverse effects of PDT include foveal damage, choroidal ischemia, RPE atrophy, and secondary choroidal NV, all of which can worsen long-term visual outcomes.^7,8^ In an effort to reduce the frequency of these complications, alterations to the standard PDT protocol to reduce damage to the RPE and underlying structures have been suggested and implemented in clinical practice. These alterations include halving the dose of verteporfin to 3 mg/m^2^ (half-dose PDT) or reducing the intensity, also termed fluence, of the laser to 25 J/cm^2^ (half-fluence PDT). Both protocols have shown comparable efficacy to standard PDT while simultaneously reducing damage to the RPE and choroidal vasculature.^9–11^ The possibility of further improvement to the safety profile of PDT exists in the form of half-dose, half-fluence PDT (half-half PDT), which uses both half-dose verteporfin (3 mg/m^2^) and half-fluence laser light (25 J/cm^2^). The current research on half-half PDT is limited to 2 published studies with small sample sizes that did not show statistically significant improvement in VA.^11,12^ The purpose of this study is to assess the efficacy of half-half PDT for the treatment of CSCR compared with other PDT protocols.

## Methods

This single-center, single-arm retrospective analysis was conducted under the tenets of the Declaration of Helsinki and approved by the Health Research Ethics Board of the University of Alberta. All patients with either acute or chronic CSCR receiving treatment with half-half PDT at Alberta Retina Consultants (Alberta, Canada) between September 2014 and July 2023 were included. Exclusion criteria included any concomitant ocular disease or previous ocular surgery, intraocular injection, or laser therapy of any form in the treated eye. All eyes in the study had a minimum of 3 months of posttreatment follow-up, and those without a 3-month follow-up visit were excluded. Eyes treated simultaneously with half-half PDT and any other treatment modality were also excluded. Symptoms were present for a minimum of 3 months before treatment was considered in all eyes. Outcomes included VA, central subfield thickness (CST), and fluid resolution assessed at 3 months along with recurrence of CSCR, need for retreatment, and reports of adverse events.

Clinical evaluation of all patients included fluorescein angiography and optical coherence tomography (OCT) with automated CST measurement along with best-corrected VA (BCVA) measurements obtained using a standard Snellen chart. Snellen VA measurements were converted to Early Treatment Diabetic Retinopathy Study (ETDRS) letters for the purposes of statistical analysis. Body surface area was calculated based on height and weight measurements, and a dosage of verteporfin of 3 mg/m^2^ was administered intravenously over the course of 10 minutes. Light at 689 nm with a fluence of 25 J/cm^2^ was then applied to the target eye for a duration of 83 seconds. The Clarion Lumenis Opal PDT was used for all treatments. The spot size was selected to cover the area of hyperfluorescence identified during angiography. In cases requiring multiple spots, an overlapping technique was used. Follow-up visits occurred 3 months after treatment and included assessment of Snellen VA, OCT image capture, and fundus examination. The primary outcome was change in VA. Reported outcomes included changes in CST and complete fluid resolution at 3 months as well as recurrence of fluid, need for retreatment, and adverse events at any timepoint after PDT. Pachychoroid NV was diagnosed in the study using a combination of fluorescein angiography, OCT, and OCT angiography.

Statistical analysis was performed using SPSS. Quantitative data were analyzed using *t* tests with generalized estimated equations correction. Binomial data were analyzed using χ^2^ tests.

## Results

A total of 378 eyes of 352 patients with CSCR naïve to treatment were included in the study. A total of 98 eyes had 6 or more months of posttreatment follow-up. The mean patient age was 50.4 years old (range, 23-86, SD, 12.6), and the study population was predominantly male (74.4%, n = 262). Use of steroid medications was noted in 31 patients (8.8%) at the time of presentation. Baseline VA was 67.2 ETDRS letters (range, 0-85, SD, 14.1). The mean time to treatment in our patient population was 4.6 months (Table 1).

**Table 1.** Baseline Characteristics of the Study Population.

Patients/eyes, no.	352/378
Mean age (y)	50.4
Male sex, no. (%)	262 (74.4)
Steroid use, no. (%)	31 (8.8)
Mean time to treatment (mo)	4.6

The most common steroid used was an inhaler for asthma in 10 of the cases. Of the remaining 21 patients, 8 were on a systemic steroid, 5 received steroid joint injections, 4 used a topical steroid cream, 2 were on a nasal spray, and 2 were using steroid eye drops. Steroids were stopped in most cases. In patients on systemic steroids, the doses were decreased but not stopped due to the underlying autoimmune diseases.

**Figure 1.**
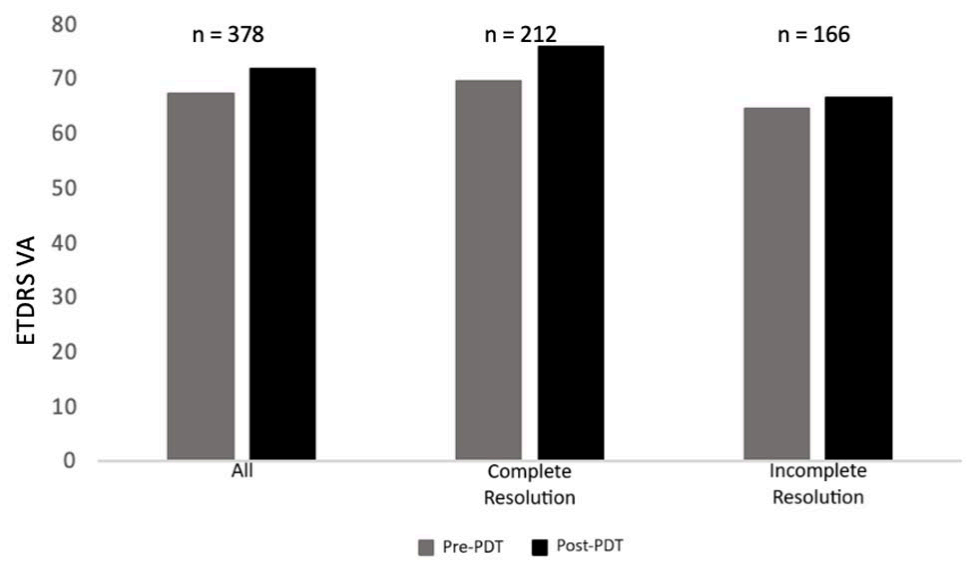
Comparison of pretreatment and posttreatment BCVA. Mean pretreatment and posttreatment mean BCVA values (ETDRS letters) are presented for the overall study population as well as for subgroups that showed both complete and incomplete fluid resolution after PDT treatment. Abbreviations: BCVA, best-corrected visual acuity; ETDRS, Early Trial Diabetic Retinopathy Study; PDT, photodynamic therapy; VA, visual acuity.

**Figure 2.**
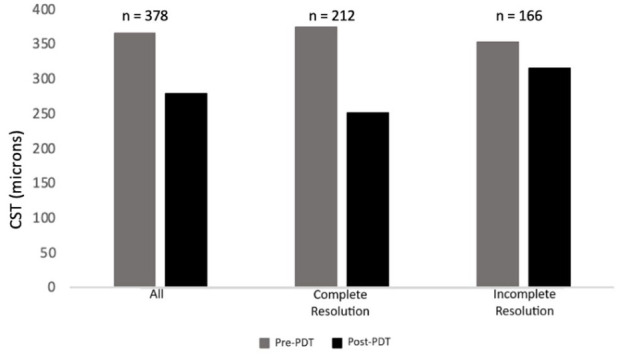
Comparison of pretreatment and posttreatment CST. Mean pretreatment and posttreatment CST values (microns) are presented for the overall study population as well as for subgroups that showed both complete and incomplete fluid resolution after PDT treatment. Abbreviations: CST, central subfield thickness; PDT, photodynamic therapy.

Statistically significant visual gains were seen within our study cohort at 3 months after PDT treatment (Figure 1). The mean visual gain was 4.4 ETDRS letters (67.2 ETDRS letters to 71.6 ETDRS letters; *P* < .05). CST also significantly improved at 3 months after PDT treatment, with a decrease of 85.8 µm (*P* < .05) (Figure 2). Complete resolution was defined as no subretinal/intraretinal fluid on OCT imaging 3 months after PDT treatment. In our cohort, this was seen in 212 eyes (56.8%). The visual improvement was significant in the complete resolution group (*P* < .05) but was not significant in the incomplete resolution group (*P* = .2). Visual gains between the complete and incomplete resolution groups were statistically significant (6.4 ETDRS letters vs 1.9 ETDRS letters; *P* < .05).

With regards to CST, the most dramatic improvement was seen in the complete resolution group, with a decrease of 122.9 µm (*P* < .05). Those with incomplete resolution had a more modest, yet still statistically significant, improvement of 37.8 µm (*P* < .05). There was a significant difference between the 2 groups when comparing the change in CST. Time to treatment after presentation was 5 months in the complete resolution group vs 3.7 months in the incomplete resolution group (*P* = .2). When extending the follow-up to 6 months, another 30 eyes had total resolution of fluid on imaging. Thus, a total of 242 eyes (64%) had complete resolution of CSCR at 6 months. In eyes with 6 months of posttreatment follow-up, the VA improved by 3.1 ETDRS letters (*P* < .05), and the CST decreased by 52.7 µm (*P* < .05).

Recurrence of CSCR was defined in our study as recurrence of fluid after complete resolution. In our cohort, 29 eyes (13.7%) had recurrence after complete resolution. The mean time to recurrence was 28 months after treatment. A total of 135 eyes in the study required retreatment at some point, including eyes that had either incomplete resolution or recurrence of fluid after complete resolution (Table 2). Most patients (44%) underwent repeat half-dose, half-fluence PDT. The mean VA after retreatment was 69.5 ETDRS letters, and there was no significant difference when comparing the different treatment modalities. Resolution of fluid was seen in 89 eyes (65.9%) that underwent retreatment.

**Table 2.** Treatment Modalities of Eyes Requiring Retreatment After Initial Half-Half PDT.

Treatment Modality	n = 135
Half-dose, half-fluence PDT, no. (%)	59 (43.7)
Half-dose, full-fluence PDT, no. (%)	46 (34.1)
Intravitreal anti-VEGF, no. (%)	28 (20.7)
Focal laser photocoagulation, no. (%)	2 (1.5)

Abbreviations: anti-VEGF, antivascular endothelial growth factor; PDT, photodynamic therapy.

No cases of macular atrophy were seen in our patient cohort after PDT. Over the course of the study, pachychoroid neovasculopathy was identified in 13 eyes (3.4%) receiving half-half PDT.

## Conclusions

This study presents the largest cohort to date of patients with CSCR undergoing half-dose, half-fluence PDT. Statistically significant improvements in VA and CST were noted along with complete resolution rates of 56.8% and 64.0% at 3 and 6 months posttreatment. The most common PDT treatment protocol currently used in the management of CSCR is half-dose, full-fluence PDT. Results from 2 recent randomized controlled trials that investigated half-dose PDT in the treatment of CSCR, PLACE and SPECTRA, showed complete resolution rates of 67.2% and 78.0%, respectively.^13,14^ Although direct comparison between studies is difficult due to differences in study design and methodology, the 6-month resolution rate reported in our study is similar to that of the previously mentioned trials. Of note, PDT spot size was not routinely documented in our patient population. The size of the spot related to OCT/fluorescein angiography may not be as accurate as indocyanine green–guided treatment, which is considered the current gold standard.^8^ In practice, we try to minimize this by ensuring a spot size larger than the area of leakage and direct comparison of the spot size with the images before treatment.

Our study reported a recurrence rate of 13.7%, while the PLACE trial reported a recurrence rate of 7.0%.^13^ The interpretation of this comparison is difficult due to differences in methodology. Our retrospective analysis included recurrences at any point after PDT treatment for which data were available, which is shown by the mean time to recurrence of 28 months. Conversely, the PLACE trial only included data up to 8 months after PDT treatment.^13^

We also found good results with repeat treatment for eyes that either did not have complete resolution or had recurrence of fluid. Some eyes in our study received antivascular endothelial growth factor (anti-VEGF) treatment for secondary treatment. There are no large clinical trials regarding anti-VEGF for treatment of CSCR. However, there are data that suggest anti-VEGF may be beneficial, especially in cases of chronic CSCR.^15,16^

The main advantage of a reduced-intensity treatment protocol like half-half PDT is hypothesized to be an improved safety profile compared with other PDT protocols. No cases of macular RPE atrophy, a potential adverse effect of PDT treatment, were reported in the present study. Although some studies have shown rates of RPE atrophy as high as 5%, no cases were reported in the PLACE trial.^13^ This brings into question whether half-dose, half-fluence PDT truly has a safety benefit compared with half-dose, full-fluence PDT. Again, our study included a larger amount of posttreatment data, while the PLACE trial was limited to 8 months.^13^ In addition, our study had a significantly larger sample size than the PLACE trial (378 vs 89 eyes), which is highly relevant considering the adverse event in question is relatively rare.^13^ The present study also noted pachychoroid NV in 3.4% of eyes, which is higher than rates reported in other studies. Again, it should be noted that the follow-up period for most patients in our cohort was significantly longer than other studies, and it is unclear whether some of these cases are the result of undertreatment or a new disease entity.

The main strength of the present study is the large cohort of treatment-naïve patients with CSCR and the long period of data collection, which allows for accurate assessment of disease recurrence and adverse events. This is of particular importance as many adverse events of PDT treatment, such as RPE atrophy, are infrequent and may not be accurately represented in a small sample of patients. Previous investigations by Park et al^11^ and Liu et al^12^ on the use of half-half PDT for CSCR were complicated by their small scale, which did not produce statistically significant findings. In the present study, we were able to provide an estimate of expected VA and anatomic improvements with half-dose, half-fluence PDT, giving further clarity to treatment options in the management of CSCR. Other limitations of this study are specific to its design as it is a single-center, single-arm retrospective analysis. Although the patients in this study were treated by multiple providers, all were treated at the same center, which limits the generalizability of the results. Additionally, as half-half PDT is the preferred treatment protocol at this center, a comparative analysis including other forms of PDT was not performed. Despite outcomes from our study being compared with others in the literature for half-dose PDT, a multi-arm study would provide for more robust evaluation of the effectiveness of half-half PDT in relation to other PDT protocols. Furthermore, both acute and chronic CSCR were included in this study, and existing literature has shown that there are differences in the pathologic characteristics and outcomes between both forms of the disease.^6^ Without performing a subgroup analysis, it is uncertain if either form is more or less responsive to treatment with half-half PDT. Additionally, determination of treatment modality was based on provider opinion, which creates the potential for selection bias. Some cases of CSCR not included in this analysis were treated with focal laser photocoagulation in addition to PDT or with other PDT protocols, such as half-dose, full-fluence. With this in mind, it should be noted that some cases of CSCR may not be amenable to treatment with half-dose, half-fluence PDT and may warrant more aggressive treatment. Last, major adverse events like choroidal NV are recorded in the electronic medical record, whereas mild adverse events, such as pain or photosensitivity, in the days after treatment are not. Therefore, it is possible that the benefits of half-half PDT related to these mild adverse events were undetected in the present study.

This study represents the largest dataset specific to half-dose, half-fluence PDT for the treatment of CSCR in the literature to date. The results show that half-half PDT is an effective and safe method of treatment for active CSCR with improvement in VA, CST, and fluid resolution. No cases of RPE macular atrophy occurred in 378 treated eyes. The treatment was slightly less effective than half-dose, full-fluence PDT for resolution of fluid and recurrence rate, suggesting that half-half PDT might be an effective first-line treatment, with half-dose, full-fluence PDT being reserved for eyes that have failed treatment. Opportunities for further research would involve prospectively designed trials that include additional testing, such as microperimetry, to more accurately determine safety improvements of half-dose, half-fluence PDT compared with other PDT protocols.
